# Novel Insights Into the Effects of Interleukin 6 Antagonism in Non–ST‐Segment–Elevation Myocardial Infarction Employing the SOMAscan Proteomics Platform

**DOI:** 10.1161/JAHA.119.015628

**Published:** 2020-06-09

**Authors:** Marc J. George, Ola Kleveland, Jorge Garcia‐Hernandez, Jutta Palmen, Ruth Lovering, Rune Wiseth, Pål Aukrust, Jorgen Engmann, Jan Kristian Damås, Aroon D. Hingorani, Lars Gullestad, Juan P. Casas, Thor Ueland

**Affiliations:** ^1^ Department of Clinical Pharmacology University College London London United Kingdom; ^2^ Clinic of Cardiology St Olavs Hospital Trondheim Norway; ^3^ Department of Circulation and Medical Imaging Norwegian University of Science and Technology NTNU Trondheim Norway; ^4^ Centre for Cardiovascular Genetics Institute of Cardiovascular Science University College London London United Kingdom; ^5^ Functional Gene Annotation, Preclinical and Fundamental Science Institute of Cardiovascular Science University College London London United Kingdom; ^6^ K.G. Jebsen Thrombosis Research and Expertise Center University of Tromsø Tromsø Norway; ^7^ Research Institute of Internal Medicine Oslo University Hospital Rikshospitalet Oslo Norway; ^8^ Institute of Clinical Medicine University of Oslo Norway; ^9^ K.G. Jebsen Centre of Inflammatory Research University of Oslo Norway; ^10^ Department of Cardiology Oslo University Hospital Rikshospitalet Oslo Norway; ^11^ Section of Clinical Immunology and Infectious Diseases Oslo University Hospital Rikshospitalet Oslo Norway; ^12^ Centre of Molecular Inflammation Research Department of Clinical and Molecular Medicine NTNU Trondheim Norway; ^13^ Department of Infectious Diseases St Olav’s Hospital Trondheim University Hospital Trondheim Norway; ^14^ K.G. Jebsen Cardiac Research Centre and Centre for Heart Failure Research University of Oslo Norway; ^15^ Institute of Health Informatics University College London London United Kingdom; ^16^ Massachusetts Veterans Epidemiology Research and Information Center (MAVERIC) Boston MA

**Keywords:** inflammation, interleukin, myocardial infarction, proteomics, Inflammation, Proteomics, Clinical Studies, Myocardial Infarction

## Abstract

**Background:**

Interleukin 6 concentration is associated with myocardial injury, heart failure, and mortality after myocardial infarction. In the Norwegian tocilizumab non–ST‐segment–elevation myocardial infarction trial, the first randomized trial of interleukin 6 blockade in myocardial infarction, concentration of both C‐reactive protein and troponin T were reduced in the active treatment arm. In this follow‐up study, an aptamer‐based proteomic approach was employed to discover additional plasma proteins modulated by tocilizumab treatment to gain novel insights into the effects of this therapeutic approach.

**Methods and Results:**

Plasma from percutaneous coronary intervention–treated patients, 24 in the active intervention and 24 in the placebo‐control arm, drawn 48 hours postrandomization were randomly selected for analysis with the SOMAscan assay. Employing slow off‐rate aptamers, the relative abundance of 1074 circulating proteins was measured. Proteins identified as being significantly different between groups were subsequently measured by enzyme immunoassay in the whole trial cohort (117 patients) at all time points (days 1–3 [7 time points] and 3 and 6 months). Five proteins identified by the SOMAscan assay, and subsequently confirmed by enzyme immunoassay, were significantly altered by tocilizumab administration. The acute‐phase proteins lipopolysaccharide‐binding protein, hepcidin, and insulin‐like growth factor‐binding protein 4 were all reduced during the hospitalization phase, as was the monocyte chemoattractant C‐C motif chemokine ligand 23. Proteinase 3, released primarily from neutrophils, was significantly elevated.

**Conclusions:**

Employing the SOMAscan aptamer‐based proteomics platform, 5 proteins were newly identified that are modulated by interleukin 6 antagonism and may mediate the therapeutic effects of tocilizumab in non–ST‐segment–elevation myocardial infarction.

**Nonstandard Abbreviations and Acronyms**
ASSAIL‐MI: Assessing the Effect of Anti‐IL‐6 Treatment in Myocardial Infarction
C5A: complement component C5a anaphylatoxin
CAD: coronary artery disease
CCL23: CC motif chemokine ligand 23
CRP: C‐reactive protein
CXCL: C‐X‐C motif chemokine ligand
EIA: enzyme immunoassay
HAMP: hepcidin antimicrobial peptide
hsTnT: high‐sensitivity troponin T
IGFBP4: insulin‐like growth factor‐binding protein 4
IL: interleukin
LBP: lipopolysaccharide‐binding protein
MI: myocardial infarction
NSTEMI: non–ST‐segment–elevation myocardial infarction
NT‐proBNP: N‐terminal pro‐B‐type natriuretic peptide
PCI: percutaneous coronary intervention
PRTN3: proteinase 3
SerpinA3: serpin family A member 3
sgp130: soluble glycoprotein 130
sIL‐6R: soluble interleukin 6 receptor
SOMAmers: slow off‐rate aptamers
UPI: UniProt ID
VEGF: Vascular endothelial growth factor
WebGestalt: WEB‐based Gene Set Analysis Toolkit

**Clinical Perspective**

**What Is New?**

- Interleukin 6 antagonism with tocilizumab in non–ST‐segment–elevation myocardial infarction reduces troponin T. However, we have hitherto been unable to identify cytokines or other molecules that could contribute to this effect.
- Employing the powerful SOMAscan proteomics platform, we newly identified 5 proteins that are modulated by interleukin 6 antagonism in non–ST‐segment–elevation myocardial infarction. The acute‐phase proteins lipopolysaccharide‐binding protein, hepcidin, and insulin‐like growth factor‐binding protein 4 were all reduced during the hospitalization phase, as was the monocyte chemoattractant C‐C motif chemokine ligand 23.
- Proteinase 3, released primarily from neutrophils, was significantly elevated.

**What Are the Clinical Implications?**

- These proteins may be secondary downstream mediators of the beneficial effect of tocilizumab.
- As the interest in targeting inflammatory cytokines such as interleukin 6 in coronary disease grows, proteomics can aid in our understanding of the results of this approach and may lead to the identification of new targets and more effective therapeutics.

Interleukin (IL) 6 is a pleotropic cytokine that plays a central role in inflammation. Using the Mendelian randomization method, we previously demonstrated that IL‐6 has a causal role in coronary artery disease (CAD),^1^ findings that have recently been replicated using the phenome‐wide association approach.^2^ High circulating IL‐6 during and immediately after acute myocardial infarction (MI) is associated with myocardial injury, heart failure, and mortality^3^ and is a predictor of mortality in unstable CAD.^4^ IL‐6 is therefore considered a potential therapeutic target in CAD and MI. Tocilizumab is a humanized anti–IL‐6 receptor antibody that binds to both membrane‐bound and soluble IL‐6 receptor (sIL‐6R) thereby antagonizing IL‐6 signaling.^5^ It is licensed to treat autoimmune disorders, such as rheumatoid arthritis, but has not yet found use in cardiovascular disease. IL‐6 is downstream of IL‐1β. IL‐1β antagonism with the monoclonal antibody canakinumab has recently been shown to reduce recurrent events and IL‐6 levels in patients with established CAD in a large phase III clinical trial,^6^ thereby firmly establishing inflammatory cytokines as a therapeutic targets in CAD.

An alternative approach to chronic anti‐inflammatory therapy in patients with CAD is short‐term anticytokine therapy after MI, which avoids the consequences of long‐term immunosuppression. The Norwegian tocilizumab non–ST‐segment–elevation MI (NSTEMI) trial, the first human study of IL‐6 blockade in MI,^7^ was a 2‐center, randomized, double‐blind, placebo‐controlled trial that enrolled 121 patients with NSTEMI. Patients received a single intravenous dose of tocilizumab 280 mg or placebo before coronary angiography. Multiple blood samples were taken during the 3 days postrandomization. The primary end point was a reduction in CRP (C‐reactive protein), a marker of the acute inflammatory response. The trial met its primary end point and also demonstrated a reduction in hsTnT (high‐sensitivity troponin T) release (a secondary end point).^7^ No safety concerns were raised. In this follow‐up study using plasma samples from the original trial, we employed an aptamer‐based proteomics approach to discover proteins modulated by the administration of tocilizumab to gain novel insights into the action of this drug in this setting.

## Methods

The data that support the findings of this study are available from the corresponding author upon reasonable request.

### Patients, Sample Collection, and Processing

Stored plasma samples from patients enrolled in the Norwegian tocilizumab NSTEMI trial included in the original trial analyses (117 patients) were used in this study. The trial was approved by the Regional Committee for Medical and Health Research Ethics of South‐Eastern Norway and the Norwegian Medicines Agency, and conducted according to the Declaration of Helsinki. All participants provided written, informed consent. Plasma was obtained from peripheral venous blood drawn into EDTA tubes as previously described.^7^ For the discovery cohort, a total of 48 percutaneous coronary intervention (PCI)–treated patients (24 from the active intervention and 24 from the placebo‐control arm) were selected at random, and plasma from patients obtained 48 hours postrandomization (during hospitalization) were analyzed with the commercially available SOMAscan assay. These patients were selected given that in the original study, a significant reduction in hsTnT was only observed in PCI‐treated patients (71% of the cohort) and the greatest difference in CRP between groups was observed 48 hours postrandomization.^7^

The SOMAscan assay is a sensitive, high‐throughput assay employing slow off‐rate aptamers (SOMAmers) that are single‐stranded DNA molecules (approximately 40 mer in length) with specific affinity for the circulating proteins of interest.^8^ The “1.3k” assay employed measures of a broad range of receptors, kinases, growth factors, and secreted intracellular and extracellular proteins.

Samples were processed at the University College London with quality‐control oversight from SomaLogic. Methods are described elsewhere.^8^ Briefly, the plasma was incubated with a mixture of biotin‐labeled SOMAmers, followed by capture of all SOMAmer‐protein complexes on streptavidin beads. After further processing, a mixture of free SOMAmers is produced that quantitatively reflects the protein concentrations in the original sample. The SOMAmers are subsequently hybridized to DNA oligonucleotides on an array. The assay signal is expressed as relative fluorescence units. SomaLogic also performs normalization to reduce intrasample bias and calibration procedures to decrease between‐sample variability. After all quality‐control procedures were completed, the relative abundance of 1074 proteins were available for each sample.

### Transformation of SOMAscan Data

To enable statistical analysis, the distribution of relative fluorescence unit values was normalized by taking the natural log. This transformation was selected as it resulted in the greatest reduction in outliers, skewness, and kurtosis, and aided interpretability as compared with other transformations under consideration (Figure S1).

### SOMAscan Validation

To assess the validity of the SOMAscan assay, the concentration of IL‐6, sIL‐6R, sgp130 (soluble glycoprotein 130), and CRP measured by other means in the placebo group of the original study were compared with measurements obtained using SOMAscan. The methods used for measurement of these proteins in the original study are described elsewhere.^7^ The linear correlation between these 4 analytes was assessed with Pearson correlation.

### Protein Discovery Using SOMAscan

Independent samples *t* tests were performed to assess the mean differences in protein measurements between patients randomized to the tocilizumab and placebo groups. Post hoc correction for multiple comparisons was performed with Bonferroni test and the Benjamini‐Hochberg procedure with false discovery rate set to 0.1. The more liberal of these approaches (Benjamini‐Hochberg) was selected given the exploratory nature of the study.

### Temporal Profile of Discovered Proteins Over the Entire Time Course

To corroborate findings from the SOMAscan platform and to understand the full temporal profile of the proteins that were modulated by tocilizumab, these proteins were measured in the Norwegian tocilizumab NSTEMI trial cohort (tocilizumab n=58, placebo n=59) over the entire time course of the study. During the trial, blood samples were drawn prerandomization and at 6 time points after the administration of drug or placebo during the first 56 hours of hospitalization (day 1: evening; day 2: morning, afternoon, and evening; and day 3: morning and afternoon). Further samples were drawn at 3 and 6 months postrandomization. Plasma was stored in aliquots at −80°C until analyzed. The proteins were measured by enzyme immunoassay (EIA) using commercially available antibodies (Table S1). A commercially available CCL23 (C‐C motif chemokine ligand 23) antibody was employed in the EIA, which recognizes both CCL23 and the splice variant Ck‐beta‐8‐1, therefore only 1 measure, encompassing both of these proteins, was made. All proteins were analyzed in duplicate in a 384‐well format using a combination of a SELMA pipetting robot (Analytik Jena AG) and a BioTek (BioTek Instruments, Inc) dispenser/washer. Absorption was read at 450 nm with wavelength correction set to 540 nm using an ELISA plate reader (BioTek). Intra‐assay and interassay coefficients were <10%.

### Statistical Analysis

The effects of tocilizumab treatment on circulating levels of these proteins over time were evaluated by the presence of a significant tocilizumab*time interaction with an ANOVA test using a mixed model with patient (intercept) as a random effect, while adjusting the fixed effects of tocilizumab treatment, time, and their interaction with baseline covariates. For these analyses, log‐transformed values for all biomarkers were used to achieve normalization/near‐normalization of distributions, and data are expressed as the back‐transformed estimated marginal mean with 95% CIs. As well as analyzing the whole cohort, a subanalysis of patients treated with PCI versus untreated was also performed.

### Interaction Analysis

To give further insight, the association between significantly regulated proteins and key variables measured in the original study was assessed. These variables include CRP, hsTnT, NT‐proBNP (N‐terminal pro‐B‐type natriuretic peptide), IL‐6, and neutrophils. Log‐transformed area under the curve values of each analyte during the acute phase (days 1–3) was used to assess associations. If no interaction with tocilizumab treatment was detected, Pearson regression coefficient for the whole population was reported. If an interaction with tocilizumab was present, Pearson regression coefficient within each group was reported.

### Functional Enrichment Analysis

In order to understand how the proteins modified by tocilizumab correlate with its wider effects on the proteome, we performed a functional enrichment analysis. This involved comparing the list of proteins altered by tocilizumab with an uncorrected *P* value <0.05 (Table S2), excluding IL‐6 and sIL‐6R as they are directly modulated by tocilizumab, and SerpinA3 (serpin family A member 3) (discordant data from SOMAscan and EIA), to the list of 1054 proteins measured by the SOMAscan assay using WebGestalt (WEB‐based Gene Set Analysis Toolkit).^9^ The analysis used Gene Ontology annotation and ontology files data^10^ accessed on January 14, 2019. We set WebGestalt to report the top 100 enriched biological processes and then applied the affinity propagation function to generate the final list. Biological processes that were not relevant, eg, “regulation of symbiosis,” were excluded. Given the exploratory nature of this analysis, a false‐discovery rate threshold was not set and enrichment was considered significant with *P*<0.05.

## Results

The 48 patients whose samples were included in the SOMAscan analysis did not differ significantly from the overall cohort, neither were there any differences between the study groups (Table 1). The mean age of the cohort was 56.8 years, 42 (87.5%) were men, 12 (25%) had diabetes mellitus, 22 (45.8%) were current smokers, and mean body mass index was 30.4 kg/m^2^. Blood pressure at baseline was 138/83 mm Hg.

**Table 1:** Patient Demographics According to Treatment Group Between the Original Study and the Subset Used for the SOMAscan Assay

	Discovery Population, N=48		Original Study, N=117	
	Placebo, n=24	Active, n=24	Placebo, n=59	Active, n=58
Risk factors baseline				
Age, mean (SD), y	56.1 (10.2)	57.4 (7.2)	60.1 (9.9)	59.8 (7.7)
Women, No. (%)	3 (12.5)	3 (12.5)	5 (8.5)	9 (15.5)
Body mass index, mean (SD), kg/m^2^	30.5 (4.8)	30.3 (3.4)	27.4 (4.4)	28.8 (3.3)
Diabetes mellitus, No. (%)	7 (29.2)	5 (20.8)	10 (16.9)	11 (19.0)
Current smoking, No. (%)	10 (41.7)	12 (50)	17 (28.8)	15 (26.3)
PCI‐treated	24 (100)	24 (100)	47 (80)	41 (71)
Systolic BP, mm Hg, mean (SD)	137.7 (16.7)	139.5 (21.7)	136.8 (18.0)	139.7 (18.1)
Diastolic BP, mm Hg, mean (SD)	81.7 (12.1)	84.8 (12.0)	80.5 (12.1)	82.9 (12.0)

BP indicates blood pressure; PCI, percutaneous coronary intervention.

### SOMAscan Validation

Correlations between the SOMAscan values and the equivalent measurements from the original study were IL‐6 (*r*=0.52 *P*=0.008), sIL‐6R (*r*=0.54 *P*=0.006), sgp130 (*r*=0.49, *P*=0.02), and CRP (*r*=0.93, *P*<0.001).

### Protein Discovery Using SOMAscan

**Figure 1:**
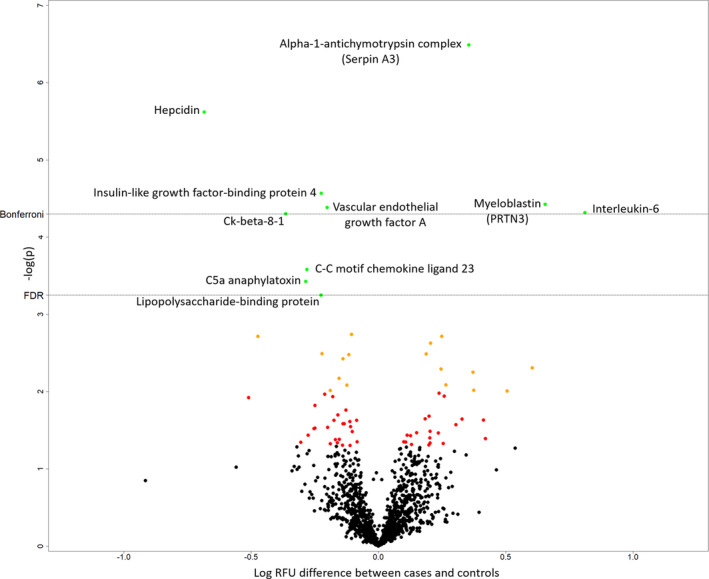
Difference in relative abundance of the 1074 SOMAscan proteins between the placebo and tocilizumab groups. Shown on the *x* axis is the log‐relative fluorescence unit (RFU) difference between groups, and on the *y* axis is the −log *P* value. Named proteins (in green) are those surpassing the false discovery rate (FDR) of 0.1 (Benjamini‐Hochberg). The Bonferroni threshold is also shown. The slow off‐rate aptamer name is used. Soluble interleukin 6 (IL‐6) receptor is beyond the axis used (*P*=2.3×10^−20^). C5A indicates complement component 5a anaphylatoxin; IGFBP4, insulin‐like growth factor‐binding protein 4; LBP, lipopolysaccharide‐binding protein; PRTN3, proteinase 3; SerpinA3, serpin family A member 3; and VEGFA, vascular endothelial growth factor A.

In the discovery cohort, 11 proteins were found to be significantly different between the tocilizumab and placebo groups using the SOMAscan assay (0.1 false discovery rate threshold of *P*=0.0005612) (Table 2, Figure 1). Two of the identified proteins were sIL‐6R (UniProt ID [UPI]: P08887) and IL‐6 (UPI: P05231), both of which had been shown to be increased by the administration of tocilizumab in the original study. Therefore, the findings for 9 of the identified proteins were novel. Of these, 2 were increased by the administration of tocilizumab. These were alpha‐1‐antichymotrypsin complex (also termed SerpinA3 [serpin family A member 3]; UPI: P01011) and myeloblastin (also termed PRTN3 [proteinase 3]; UPI: 24158).

**Table 2:** Significant Proteins Identified by the SOMAscan Assay, Ordered by *P* Value

Protein (SOMAscan Name)	Log Mean Difference Tocilizumab‐Placebo (SE)	Unadjusted *P* Value	95% CI		Bonferroni *P* Value	Benjamini‐Hochberg *P* Value
IL‐6 receptor subunit α	1.25 (0.08)	2.31E‐20	1.09	1.41	3.05E‐17	2.4809E‐17
α1‐Antichymotrypsin complex/SerpinA3	0.36 (0.06)	3.25E‐07	0.24	0.47	0.0004	0.0002
Hepcidin	−0.68 (0.13)	2.41E‐06	−0.94	−0.43	0.0032	0.0009
IGFBP4	−0.22 (0.05)	2.71E‐05	−0.32	−0.13	0.0357	0.0073
Myeloblastin/PRTN3	0.65 (0.14)	3.76E‐05	0.36	0.95	0.0496	0.0081
VEGFA	−0.20 (0.04)	4.13E‐05	−0.29	−0.11	0.0544	0.0074
IL‐6	0.81 (0.18)	4.82E‐05	0.45	1.18	0.0635	0.0074
Ck‐beta‐8‐1	−0.36 (0.08)	5.00E‐05	−0.53	−0.20	0.0658	0.0067
CCL23	−0.28 (0.07)	0.0003	−0.42	−0.14	0.3448	0.0312
C5A	−0.29 (0.07)	0.0004	−0.44	−0.14	0.4950	0.0404
LBP	−0.23 (0.06)	0.0006	−0.35	−0.10	0.7392	0.0548

Benjamini‐Hochberg false discovery rate set to 0.1. C5A indicates complement component C5a anaphylatoxin; CCL23, C‐C motif chemokine ligand 23; IGFBP4, insulin‐like growth factor‐binding protein 4; IL, interleukin; LBP, lipopolysaccharide‐binding protein; PRTN3 proteinase 3; SerpinA3, serpin family A member 3; VEGF, vascular endothelial growth factor A.

Seven proteins were decreased in the tocilizumab group as compared with the placebo group including the acute‐phase proteins HAMP (hepcidin antimicrobial peptide) (UPI: P81172), IGFBP4 (insulin‐like growth factor‐binding protein 4) (UPI: P22692), and LBP (lipopolysaccharide‐binding protein) (UPI: P18428), as well as VEGFA (vascular endothelial growth factor A) (UPI: P15692), C5A (complement component C5a anaphylatoxin) (UPI: P01031), and both the CCL23 (UPI: P55773) and its splice variant Ck‐beta‐8‐1. Table S2 lists all proteins whose relative abundance differed between groups with an uncorrected *P* value <0.05.

### Temporal Profile of Discovered Proteins Over the Entire Time Course Measured by EIA

**Figure 2:**
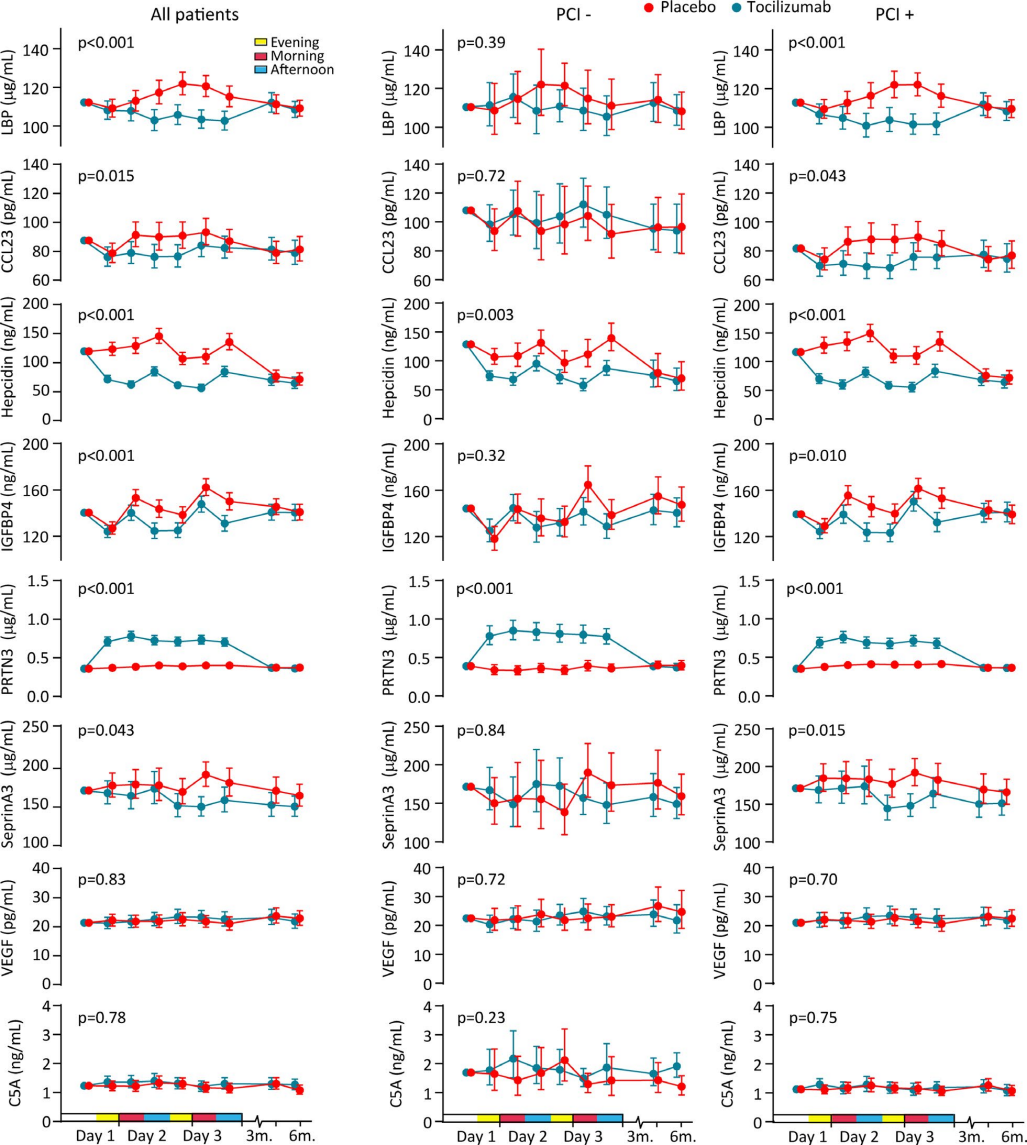
Plasma concentrations of proteins discovered by SOMAscan measured by enzyme immunoassay in the whole study cohort over time. Plasma levels of LBP (lipopolysaccharide‐binding protein), CCL23 (C‐C motif ligand 23) (cross‐reacts with Ck‐beta‐8‐1), HAMP (hepcidin antimicrobial peptide), IGFBP4 (insulin‐like growth factor‐binding 4), PRTN3 (proteinase 3), SerpinA3 (serpin family A member 3), VEGF (vascular endothelial growth factor A), and C5A (complement component 5a anaphylatoxin) during hospitalization (baseline to 3 days) and follow‐up (3 and 6 months) in patients with non–ST‐segment–elevation myocardial infarction receiving placebo (n=59) or tocilizumab (n=58) and analyzed according to whether the patient did (n=88) or did not (n=29) undergo percutaneous coronary intervention (PCI) (n=88). Circles and bars represent estimated marginal means and 95% CIs normalized for baseline values. The *P* value represents the effect of treatment.

Over the first 56 hours of serial samples during hospitalization, 6 of the 8 proteins (Ck‐beta‐8‐1 included in the CCL23 EIA) were found to have significant differences in concentration between the tocilizumab and placebo groups when assessed by EIA (Figure 2). LBP, HAMP, and IGFBP4, as well as CCL23, were reduced throughout, in agreement with the SOMAscan assay. A total of 41 of the 58 patients (70.7%) in the tocilizumab group underwent PCI, and subanalysis showed that the reductions in LBP, IGFBP4, and CCL23 were only present in this group (Figure 2).

As found with the SOMAscan assay, tocilizumab induced a rapid increase in PRTN3 that was sustained for 56 hours, compared with no changes in the placebo group (Figure 2). This effect was seen regardless of whether the patients underwent PCI (Figure 2).

In contrast to the proteomic data, however, the concentration of SerpinA3 as measured by EIA was decreased, rather than elevated, and there was no significant difference in the concentrations of VEGFA or C5A levels between the groups (Figure 2). Levels of all examined proteins were similar in the 2 groups at the 2 long‐term follow‐up time points (3 and 6 months).

### Associations of Discovered Proteins With Key Variables

**Figure 3:**
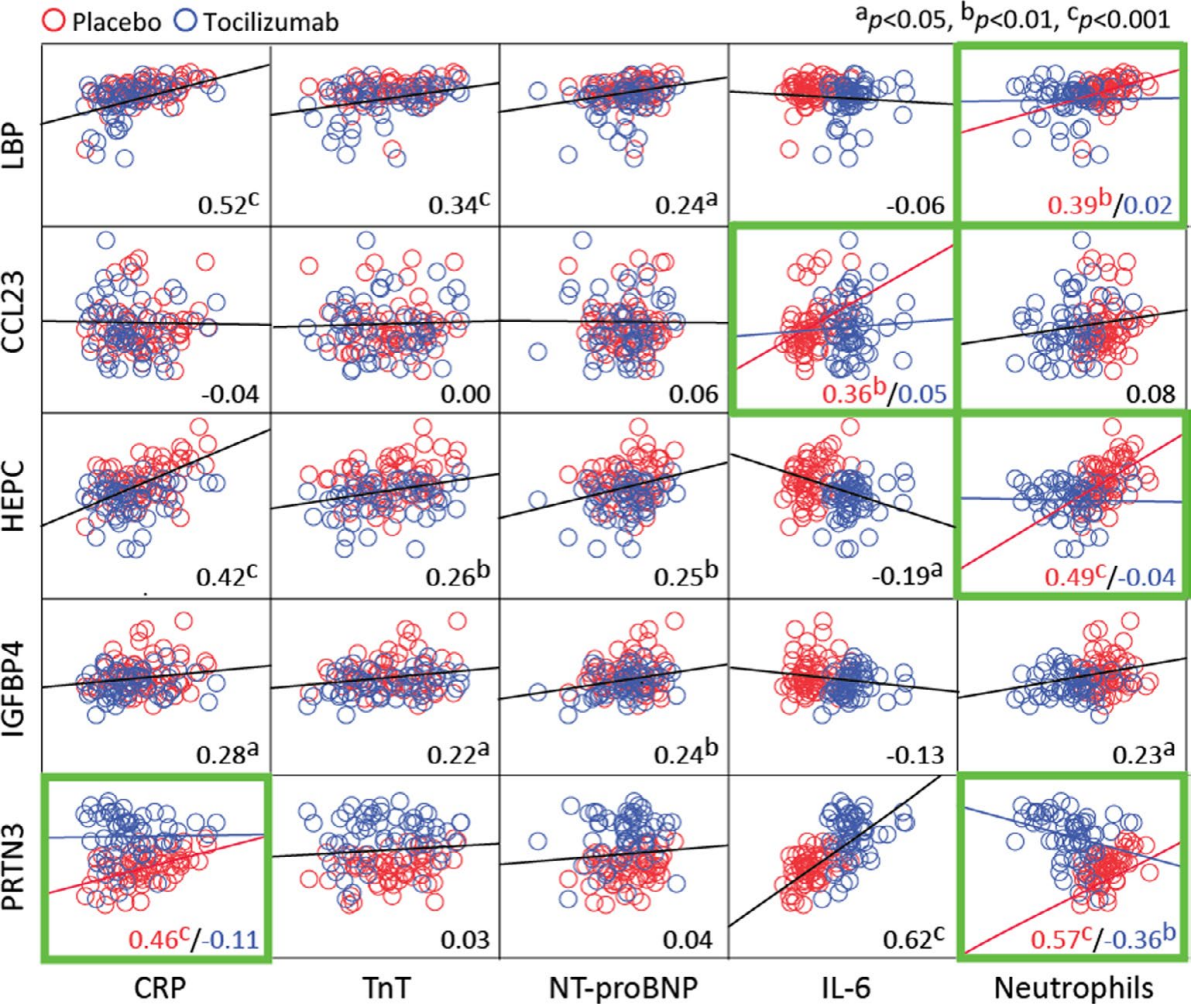
Associations between newly identified proteins modulated by tocilizumab and parameters measured in the original study. Dotplots showing associations between area under the curve (log‐transformed) of plasma levels of LBP (lipopolysaccharide‐binding protein), CCL23 (C‐C motif ligand 23), hepcidin, IGFBP4 (insulin‐like growth factor‐binding 4), and PRTN3 (proteinase 3) with CRP (C‐reactive protein), hsTnT (high‐sensitivity troponin T), NT‐proBNP (N‐terminal pro‐B‐type natriuretic peptide), interleukin 6 (IL‐6), and neutrophil counts during hospitalization (baseline to 3 days) in patients with non–ST‐segment–elevation myocardial infarction receiving placebo (red circles, n=59) or tocilizumab (blue circles n=58). Black lines represent the regression line and the numbers in the bottom right the correlation coefficient for the whole group. If an interaction with tocilizumab was detected (green frames, see Statistical Analysis), the regression lines and numbers reflect either placebo (red) or tocilizumab (blue).

There were significant positive, but relatively modest, correlations found between CRP, hsTnT and NT‐proBNP *and* LBP, HAMP, and IGFBP4; however, there was no effect of tocilizumab on these associations (Figure 3). In contrast, there was an association between CRP and PRTN3 in the placebo group, which was lost in the tocilizumab group. CCL23 was not associated with any of the variables, and PRTN3 was not associated with the myocardial markers hsTnT and NT‐proBNP.

Whereas IL‐6 was positively correlated with PRTN3, it was negatively correlated with HAMP, with the same pattern in both the placebo and tocilizumab groups (Figure 3). In contrast, a significant positive correlation between IL‐6 and CCL23 was lost in the tocilizumab group.

The strongest correlations were found with neutrophil numbers. Circulating neutrophil concentrations were positively correlated with the 3 acute‐phase proteins (LBP, HAMP, and IGFBP4) in the placebo groups (Figure 3). However, in the case of LBP and HAMP, this association was lost in the tocilizumab group. Interestingly, a significant positive correlation in the placebo group between neutrophils and PRTN3 was inversed in the tocilizumab group.

As LBP has been shown to be associated with high‐density lipoprotein cholesterol levels^11^ and tocilizumab has been shown to have a pronounced effect on lipids and in particular triglycerides,^12^ we evaluated associations between LBP and high‐density lipoprotein cholesterol. First, we did not observe an effect of tocilizumab on triglycerides (area under the curve median 4.97 versus 5.00 mmol/L, versus placebo, respectively; *P*=0.80). Second, no correlation between the area under the curves of LBP and high‐density lipoprotein cholesterol in the whole population (*r*=–0.09, *P*=0.34) at any individual time point (all *P*>0.2) or for change from baseline to 48 hours postrandomization (*r*=0.11, *P*=0.25) was observed. Evaluating these correlations within groups, ie, placebo versus tocilizumab or PCI versus non‐PCI, revealed similar results.

### Functional Enrichment Analysis

Employing WebGestalt we identified of 17 biological processes enriched by tocilizumab (Table 3). Of these enriched processes, notable ones including at least 1 of the 5 novel proteins we had identified were those related to acute inflammation such as response to tumor necrosis factor (CCL23, HAMP), cell chemotaxis (LBP, CCL23), and granulocyte migration (LBP, CCL23, PR3), as well as cardiac muscle development (HAMP).

**Table 3:** Biological Process Gene Ontology Terms Associated With the List of Proteins Differentially Expressed Following Tocilizumab Treatment

Biological Process	Enrichment Ratio	*P* Value	N	n	Proteins
Erythrocyte homeostasis	5.325	0.00051983	20	6	JAK2, EPO, GPI, VEGFA, TGFBR3, MAPK14
Cellular response to chemical stimulus	1.4005	0.00073273	545	43	Not listed given large number. Includes CCL23, LBP, PR3, HAMP
Response to TNF	2.5927	0.00079303	89	13	CCL23, HAMP, CCL24, JAK2, LTA, CXCL8, TNFRSF1A, TNFSF8, LTB, MAPK14, CCL4L1, CPNE1, CXCL16
Cell chemotaxis	2.2756	0.0012316	117	15	CCL23, LBP, CCL24, FGF18, C5, S100A9, PDGFR8, CXCL8, VEGFA, AZU1, IL‐16, MAPK14, CCL4L1, CXCL16, IL‐37
Cardiac muscle tissue development	3.4634	0.0013910	41	8	HAMP, PDGFRB, VEGFA, BMPR1A, MAP2K4, FHF8, TGFBR3, MAPK14
Induction of positive chemotaxis	7.1	0.0014841	10	4	CXCL8, VEGFA, AZU1, IL‐16
Coronary vasculature morphogenesis	10.65	0.0015702	5	3	PDGFRB, VEGFA, TGFBR3
Cardiac muscle cell development	6.4545	0.0022344	11	4	HAMP, PDGFRB, VEGFA, MAP2K4
Positive regulation of organ growth	4.9306	0.0023334	18	5	HAMP, BMPR1A, FGF8, TGFBR3, MAPK14
Monocyte differentiation	6.6563	0.0077796	8	3	APCS, CSF1R, VEGFA
Positive regulation of macromolecule biosynthetic process	1.7364	0.0088122	184	18	LBP, JAK2, EPO, PDGFRB, H2AFZ, VEGFA, TNFRSF1A, AZU1, CCNA2, CDK2, TNFSF8, BMPR1A, MAP2K4, LTB, CAMK1, MAPK14, HJV, IL‐17F
Positive regulation of tyrosine phosphorylation of STAT protein	2.8239	0.0093213	44	7	JAK2, CRLF1, EPO, CSF1R, VEGFA, TNFRSF1A, CLCF1
Granulocyte migration	2.3843	0.0099917	67	9	CCL23, LBP, PRTN3, CCL24, S100A9, CXCL8, MAPK14, CCL4L1, IL37
Aorta morphogenesis	5.9167	0.011205	9	3	PDGFRB, BMPR1A, FGF8
Regulation of nitrogen compound metabolic process	1.2723	0.012931	572	41	Not listed given large number. Includes CCL23, LBP, PRTN3, HAMP, IGFBP4
Cell migration	1.4604	0.014433	316	26	Not listed given large number. Includes CCL23, LBP, PRTN3
Negative regulation of neuron death	2.485	0.018581	50	7	JAK2, CRLF1, EPO, GPI, MAP2K4, FGF8, CLCF1

Seventeen gene ontology biological processes were identified as enriched in the blood in patients following tocilizumab treatment. AZU1 indicates azurocidin 1; BMPR1A, bone morphogenetic protein receptor 1A; BMPR1A, bone morphogenic protein receptor type 1A; CAMK1, calcium/calmodulin‐dependent protein kinase 1; CCL23, C‐C motif chemokine ligand 23; CCL24, C‐C motif chemokine ligand 24; CCL4L1, C‐C motif chemokine ligand 4‐like 1; CCNA2, cyclin A2; CDK2, cyclin‐dependent kinase 2; CLCF1, cardiotropin‐like cytokine factor 1; CRLF1, cytokine receptor‐like factor 1; CSF1R, colony‐stimulating factor 1 receptor; CXCL8, C‐X‐C motif chemokine ligand 8; EPO, erythropoietin; FGF8, fibroblast growth factor 8; GPI, glycosylphosphatidylinositol; H2AFZ, H2A histone family, member Z; HAMP, hepcidin antimicrobial peptide; HJV, hemojuvelin; IGFBP4, insulin‐like growth factor‐binding protein 4; IL17F, interleukin 17F; IL37, interleukin 37;JAK2, Janus kinase 2; LBP, lipopolysaccharide‐binding protein; LTB, lymphotoxin β; MAP2K4, mitogen‐activated protein kinase 4; MAPK14, mitogen‐activated protein kinase 14; N, number of proteins associated with gene ontology term in SOMAscan assay data set; n, number of proteins associated with gene ontology term in SOMAscan assay data set and the query list of differentially expressed proteins identified following tocilizumab treatment; PDGFRB, platelet‐derived growth factor receptor β; PRTN3, proteinase 3; S100A9, S100 calcium‐binding protein A9; TNFRSF1A, tumor necrosis factor receptor super family 1A; TNFSF8, tumor necrosis factor superfamily member 8; and VEGFA, vascular endothelial growth factor A.

## Discussion

Employing an aptamer‐based proteomics approach on samples from the Norwegian tocilizumab NSTEMI trial cohort together with EIA measurements of these proteins across all time points, we have identified 5 proteins that are modulated by the administration of tocilizumab in NSTEMI. These results have revealed several interesting insights into the effects of tocilizumab treatment in NSTEMI and raised some important questions.

### Acute‐Phase Proteins: LPB, HAMP, IGFBP4—Potential Secondary Mediators of IL‐6 Inhibition During NSTEMI

MI is associated with an acute‐phase response, driven largely by IL‐6 signaling.^13^ Thus, we have recently demonstrated that IL‐6 blockade markedly lowers CRP in NSTEMI.^7^ Given this, it is not surprising that the SOMAscan assay identified other acute‐phase proteins (LBP, HAMP, and IGFBP4), which were lower in the tocilizumab treatment group and were positively correlated with CRP. Furthermore, while a positive correlation was detected between LBP and HAMP *and* neutrophil numbers in the placebo group, this association was not present in patients treated with tocilizumab, which suggests that the decline in these markers could be directly linked to the neutropenia observed during anti–IL‐6 therapy. Nonetheless, apart from reflecting an attenuating effect of IL‐6 antagonism on the acute‐phase response, the downregulation of these molecules could also have a direct physiological impact.

HAMP is a key regulator of iron homeostasis and has recently been shown to be produced within the myocardium post‐MI by both macrophages^14^ and cardiomyocytes.^15^ IL‐6 has also been shown to induce monocyte gene expression of HAMP^14^ and may be responsible for its production in myocytes.^15^ Interestingly, in the functional analysis, HAMP clustered with other proteins involved in cardiac muscle development, and indeed there is increasing evidence that HAMP plays a central role in controlling myocyte iron and oxygen redox status and thus maintaining normal metabolism.^16^ Given this, its reduction post‐MI may impact the ability of infarcted myocardium to control oxygen free radicals produced by iron overload.^17^ However, conversely, HAMP has also been shown to contribute to plaque destabilization via activation of macrophages,^18^ and therefore there may also be advantages to its downregulation post‐MI.

LBP, which plays a key role in the immune response to gram‐negative bacteria, has also recently been identified as a novel component of atherosclerotic plaques that is released into the circulation after plaque rupture^19^ and is associated with increased cardiovascular risk.^20^ LBP has been shown to closely associate with high‐density lipoprotein cholesterol^11^ and tocilizumab has pronounced effects on lipids,^12^ but we were unable to demonstrate any associations between tocilizumab, LBP, and lipids. In the functional analysis, LBP was associated with multiple enriched biological processes including cell and granulocyte chemotaxis/migration, suggesting that it may have a significant role in mediating inflammation post‐MI. Indeed, it has been suggested that LBP may have a direct role in myocardial inflammation post‐MI via CD14/Toll‐like receptor 4–associated pathways.^19^

IGFBP4, a binding protein that prolongs the half‐life of insulin‐like growth factor, may also play a role in acute MI. Insulin‐like growth factor stimulation of macrophages results in inflammatory cytokine release^21^ and it has also been shown to play a role in plaque development.^22^

Therefore, given the evidence from the functional analysis and emerging literature, it is possible that the reduction in the 3 identified acute‐phase proteins may contribute to the anti‐inflammatory effects of IL‐6 inhibition in NSTEMI.

### CCL23: a Monocyte Chemoattractant That Could Contribute to the Anti‐Inflammatory Effect of Tocilizumab in NSTEMI

CCL23 is a member of the CC chemokine family and is involved in leukocyte trafficking and activation. For example, it has been shown to act as both a monocyte chemoattractant and to stimulate the release of monocyte chemoattractant protein‐1 and tumor necrosis factor α from these cells.^23^ A specific role for CCL23 in ischemic heart disease is supported by increased expression in atherosclerotic lesions and in the circulation in patients with atherosclerosis,^23^ and by its association with cardiovascular outcomes.^20^ However, its role in MI has not previously been studied and this is the first study to our knowledge to suggest a direct link between IL‐6 signaling and CCL23. Given that monocytes play a key role in the inflammatory response during plaque destabilization and post‐MI maladaptive remodeling,^24^ the suppression of CCL23 may be a key mechanism by which tocilizumab beneficially modifies the inflammatory milieu post‐MI and warrants further investigation.

However, although CCL23 was associated with several enriched biological processes related to inflammation, including response to tumor necrosis factor, cell chemotaxis/cell migration, and granulocyte migration, IL‐6 inhibition had a variable effect on proteins associated with these processes. For example, with regards to cell chemotaxis (Table S3), certain proteins were downregulated in the same manner as CCL23, including the cytokine IL‐37 and chemokine CXCL (C‐X‐C motif chemokine ligand) 16. However, others were elevated, such as the cytokine/chemokines CXCL8, CCL24, CCL4L1, and IL‐16. Given this, tocilizumab likely has pleiotropic effects on chemotaxis post‐MI and the overall net effect on cell trafficking is therefore difficult to discern.

### PRTN3 is Related to Neutropenia During Tocilizumab Therapy in NSTEMI

A major finding in our study was the marked and early increase in PRTN3 during IL‐6 inhibition. PRTN3 is a serine protease mainly expressed by neutrophils, but may also be expressed by endothelial cells.^25^ The associated proteases myeloperoxidase and azurocidin are also elevated in the SOMAscan assay but did not reach the multiplicity adjusted threshold of significance (Table S2). Although the mechanism for tocilizumab‐induced neutropenia is not firmly established, it may occur rapidly within few hours, indicating limited involvement of bone marrow.^26^ In patients with rheumatoid arthritis, the neutropenia induced by tocilizumab has been attributed to the inhibition of antiapoptotic actions of IL‐6 on activated neutrophils,^27^ and evidence for a proapoptotic effect of tocilizumab treatment has been demonstrated in vitro.^28^ Thus, the rapid increase in PRTN3 could be related to its release from apoptotic neutrophils. This is supported by the interaction analysis, which revealed that while neutrophils and PRTN3 were positively correlated in the placebo group, they were negatively correlated in the tocilizumab group. In addition, PRTN3 itself may also directly stimulate neutrophil apoptosis,^29^ which may further increase PRTN3 release. As neutrophil apoptosis is a key process in the resolution of inflammation, we speculate that the proapoptotic effects of PRTN3 on neutrophils could contribute to the beneficial effects of tocilizumab following MI.

As with other neutrophil serine proteases, however, there are also many potential deleterious effects associated with its release in the context of MI.^30^, ^31^ Furthermore, antibodies against PRTN3 and myeloperoxidase are associated with antineutrophil cytoplasmic antibody–related vasculitis.^32^ Thus, this important and interesting finding warrants further investigation in order to establish the underlying mechanism and to understand whether the increased levels of PRTN3 may augment or mitigate the beneficial effects of IL‐6 inhibition in NSTEMI. From a clinical perspective, it would also be important to assess whether there is an increased risk of developing antineutrophil cytoplasmic antibody autoantibodies in patients who receive tocilizumab in the context of MI.

### Is IL‐6 Antagonism Only Likely to Be of Benefit in Patients With Reperfused MI?

In the Norwegian tocilizumab NSTEMI trial, the area under the curve for hsTnT during hospitalization was significantly lower in the tocilizumab arm (159 versus 234 ng/L per h, *P*=0.07); however, subanalysis of the study demonstrated that this reduction was only observed in the group of patients who underwent PCI.^7^ Similarly, in this follow‐up study, the reductions in LBP, IGFBP4, and CCL23 were also only observed in the group that underwent PCI. These data suggest that IL‐6 antagonism may have more significant anti‐inflammatory and therapeutic effects in the setting of reperfusion. This is possibly because reperfusion is associated with a substantially more robust inflammatory response compared with ischemia alone,^33^ characterized by both increased myocardial IL‐6 mRNA transcription^34^ and plasma concentrations.^35^ The ASSAIL‐MI (Assessing the Effect of Anti‐IL‐6 Treatment in Myocardial Infarction) trial,^36^ which has enrolled patients with ST‐segment–elevation MI undergoing primary PCI, will help determine whether targeting IL‐6 in the context of early reperfusion is likely to be of therapeutic benefit.

### Study Strengths and Limitations

Our study has several strengths including the application of a proteomics platform with over 1000 analytes, corroboration of the assay with proteins measured in the original study, and verification of SOMAscan findings by EIA across the whole time course. However, there are some notable limitations and caveats.

Although significant, the correlation between EIA and SOMAscan measures of IL‐6, sIL‐6R, and sgp130 were relatively weak (*r*=0.49–0.52), whereas the correlation between measures of CRP were strong (*r*=0.93). This may reflect the accuracy and sensitivity of assays used for these analytes in the original study. Whereas CRP was the primary outcome and measured with a high‐sensitivity, automated, clinical‐grade immunoturbidimetric assay (Roche Diagnostics) with a within‐run coefficient of variance of ≈1.5% and between‐run coefficient of variance of 2.5%^37^ the other assays were research‐grade EIAs (R&D Systems), which are less accurate and have higher within‐ and between‐run coefficient of variance values of up to 10%.^38^

Furthermore, although 8 of the 11 proteins that were identified by the SOMAscan proteomics platform were confirmed to be significantly altered by tocilizumab over the entire study time course by EIA, this was not the case for SerpinA3, VEGFA, and C5A. This may be attributable to the time point selected for SOMAscan not being reflective of the entire time course, technical variability in the assays employed, or biological instability of some of the measured analytes.^39^

We only performed the initial SOMAscan assay on samples from 48 patients (24 tocilizumab, 24 controls) at a single time point. Given the small sample size, and the liberal adjustment for multiplicity that was selected (Benjamini‐Hochberg with a false discovery rate set to 0.1), there is a chance that we selected false‐positive proteins for further study. Conversely, given the small sample size, there may be several important false‐negative proteins within this group that are significantly regulated by IL‐6, and increasing the number of samples would have given us greater power to identify them. Furthermore, the use of samples from the patients who underwent PCI for the initial proteomics may have biased the discovery of proteins towards those that are only modulated in this context. The list of 50 proteins that were different between the 2 groups with an uncorrected *P* value <0.05 is included in Table S2. Our study did not have any hard outcome measures and we are not proposing that any of the markers represent prognostic biomarkers in NSTEMI.

Finally, to perform the network analysis using WebGestalt, we included all of the proteins, which were significantly altered by tocilizumab, including those that were not significant after adjustment for multiplicity. Furthermore, these proteins were not subsequently measured in the whole cohort by EIA. Therefore, the results of the network analysis must be considered complementary and hypothesis‐generating and further work is required to understand the effects of IL‐6 inhibition on the wider proteome post‐MI.

## Conclusions

We have hitherto been unable to identify cytokines or other molecules that could contribute to the benefit of tocilizumab in our NSTEMI study.^40^, ^41^ Herein, however, by employing a powerful, aptamer‐based proteomics platform, we have identified several proteins that are significantly modulated by the administration of tocilizumab in patients with NSTEMI, particularly in the context of PCI, and that could potentially be secondary downstream mediators of the beneficial effect of tocilizumab in this cohort. The findings with regard to PRTN3 and CCL23 are particularly noteworthy and warrant further investigation. As the interest in targeting inflammatory cytokines in CAD grows, understanding the consequence of this approach in detail may lead to the identification of new targets and more effective therapeutics.

## Sources of Funding

This study was funded by the National Institute for Health Research (NIHR) University College London Hospitals Biomedical Research Centre. MJG was funded by a Wellcome Trust Clinical Research Training Fellowship during the project. ADH is an NIHR senior investigator.

## Disclosures

None.

## Supporting information


**Tables S1–S3 Figure S1**
