# Supplementary material for: Novel Insights Into the Effects of Interleukin 6 Antagonism in Non–ST‐Segment–Elevation Myocardial Infarction Employing the SOMAscan Proteomics Platform
Source: J Am Heart Assoc. 2020 Jun 9;9(12):e015628. doi: 10.1161/JAHA.119.015628 (PMC7429051; doi:10.1161/JAHA.119.015628)
Supplement: Supplementary file 1 — Tables S1–S3 Figure S1 [file JAH3-9-e015628-s001.pdf]

Supplemental Material

Table S1. Antibodies used for EIA.

Protein	Supplier	Antibody Capture	Antibody Detection
Lipopolysaccharide-binding protein	R&D systems	Mouse, monoclonal	Goat, polyclonal
C-C motif chemokine ligand 23	R&D systems	Mouse, monoclonal	Goat, polyclonal
Hepcidin	R&D systems	Rabbit, monoclonal	Rabbit, monoclonal
Insulin like growth factor binding protein 4	R&D systems	Mouse, monoclonal	Goat, polyclonal
Vascular endothelial growth factor A	R&D systems	Mouse, monoclonal	Goat, polyclonal
Myeloblastin/PRTN3	R&D systems	Mouse, monoclonal	Mouse, monoclonal
Alpha-1-antichymotrypsin complex/Serpin A3	Sino Biological	Rabbit, monoclonal	Rabbit, monoclonal

Table S2. All proteins identified by the SOMAscan assay with a p-value of <0.05.

**Protein (SOMAscan name)**	**Log Mean diff TCZ-placebo**	**SE**	**p value**
**Interleukin-6 receptor subunit alpha**	1.25	0.08	2.31E-20
**Alpha-1-antichymotrypsin complex**	0.36	0.06	3.25E-07
**Hepcidin**	-0.68	0.13	2.41E-06
**Insulin-like growth factor-binding protein 4**	-0.22	0.05	2.71E-05
**Myeloblastin**	0.65	0.14	3.76E-05
**Vascular endothelial growth factor A**	-0.20	0.04	4.13E-05
**Interleukin-6**	0.81	0.18	4.82E-05
**Ck-beta-8-1**	-0.36	0.08	5.00E-05
**C-C motif chemokine 23**	-0.28	0.07	0.000262
**C5a anaphylatoxin**	-0.29	0.07	0.000376
**Lipopolysaccharide-binding protein**	-0.23	0.06	0.000561
C-type lectin domain family 4 member M	-0.11	0.03	0.001810
Mitogen-activated protein kinase 14	0.25	0.08	0.001927
Azurocidin	0.20	0.06	0.002362
Protein S100-A9	-0.22	0.07	0.003216
Kin of IRRE-like protein 3	0.19	0.06	0.003261
Growth arrest-specific protein 1	-0.12	0.04	0.003322
Complement decay-accelerating factor	-0.14	0.05	0.003765
Macrophage colony-stimulating factor 1 receptor	0.25	0.08	0.005122
Tyrosine-protein kinase JAK2	-0.15	0.05	0.006712
C-C motif chemokine 4-like	0.27	0.10	0.008218
Serum amyloid P-component	-0.12	0.04	0.008226
Glucose-6-phosphate isomerase	0.37	0.14	0.009625
Interleukin-17F	-0.19	0.07	0.009714
Histone H2A.z	0.51	0.19	0.009800

Immunoglobulin gamma Fc region receptor III-B	0.24	0.09	0.010483
Cerebral dopamine neurotrophic factor	-0.21	0.08	0.010806
Myeloperoxidase	0.26	0.10	0.011464
Tumor necrosis factor receptor superfamily member 1A	-0.18	0.07	0.011650
Cystatin-S	-0.25	0.10	0.015080
Somatostatin-28	-0.13	0.05	0.017296
Bone morphogenetic protein receptor type-1A	-0.16	0.07	0.020002
Interleukin-16	0.20	0.08	0.020767
Interleukin-8	0.18	0.08	0.022531
Copine-1	0.33	0.14	0.022640
beta-adrenergic receptor kinase 1	0.41	0.18	0.023288
Inter-alpha-trypsin inhibitor heavy chain H4	-0.09	0.04	0.023431
Hemojuvelin	-0.17	0.07	0.023521
Dynactin subunit 2	-0.11	0.05	0.024462
Transforming growth factor beta receptor type 3	-0.13	0.06	0.026103
Ephrin-A4	-0.14	0.06	0.026112
C-X-C motif chemokine 16	-0.11	0.05	0.028414
Dual specificity mitogen-activated protein kinase kinase 4	-0.20	0.09	0.029091
Erythropoietin	-0.25	0.11	0.029717
Moesin	-0.25	0.11	0.030028
Cardiotrophin-like cytokine factor 1 Complex	0.20	0.09	0.032515
L-lactate dehydrogenase B chain	0.24	0.11	0.034243
Lymphotoxin alpha1:beta2	0.11	0.05	0.036623
Platelet-derived growth factor receptor beta	-0.28	0.13	0.036766
Cyclin-dependent kinase 2:Cyclin-A2 complex	0.20	0.10	0.039800
Fibroblast growth factor 18	0.42	0.20	0.040492
Tropomyosin beta chain	-0.15	0.07	0.041355
Proteasome subunit alpha type-1	0.10	0.05	0.044684
Complement component C6	-0.08	0.04	0.044729
Tumor necrosis factor ligand superfamily member 8	0.11	0.05	0.044754

Protein FAM107A	-0.16	0.08	0.045972
Fibroblast growth factor 8 isoform A	0.20	0.10	0.046155
C-C motif chemokine 24	0.25	0.12	0.046868
Calcium/calmodulin-dependent protein kinase type 1	-0.19	0.09	0.047212
Desert hedgehog protein N-product	0.20	0.10	0.049036
Interleukin-37	-0.11	0.06	0.049805

Table S3. Proteins associated with the enriched GO term ‘cell chemotaxis’.

Protein	Effect of TCZ	Protein class
**Cell Chemotaxsis**		
C-C motif chemokine ligand 23 (CCL23)	Reduced	Chemokine
LPS binding protein (LBP)	Reduced	Acute Phase Protein
Complement C5 (C5)	Reduced	Complement
S100 calcium binding protein A9 (S100A9)	Reduced	Calcium-binding protein
Platelet derived growth factor receptor beta (PDGFRB)	Reduced	Growth factor
Vascular Endothelial Growth factor A (VEGFA)	Reduced	Growth Factor
Interleukin 37 (IL37)	Reduced	Cytokine
C-X-C motif chemokine ligand 16 (CXCL16)	Reduced	Chemokine
Fibroblast growth factor 18 (FGF18)	Elevated	Growth Factor
Azurocidin 1 (AZU1)	Elevated	Serine Protease
C-C motif chemokine ligand 24 (CCL24)	Elevated	Chemokine
C-X-C motif chemokine ligand 8 (CXCL8)	Elevated	Chemokine
C-C motif chemokine ligand 4 like 1 (CCL4L1)	Elevated	Chemokine
Mitogen-activated protein kinase 14 (MAPK14)	Elevated	Kinase
Interleukin 16 (IL16)	Elevated	Chemokine

Figure S1. Selection of SOMAscan data transformation method. Proteomic transformations assessing the distribution of the **a)** proportion of outliers **b)** skewness and **c)** kurtosis. This was performed with data from all proteins in the 1.3k SOMAscan assay (before proteins removed in the quality-control process). The X axis refers to the 1.3k SOMAscan proteins. The Y axis denotes, from top to bottom to the proportion of outliers, skewness and kurtosis. Vertical red line means the division of half of total number of proteins (N=659).

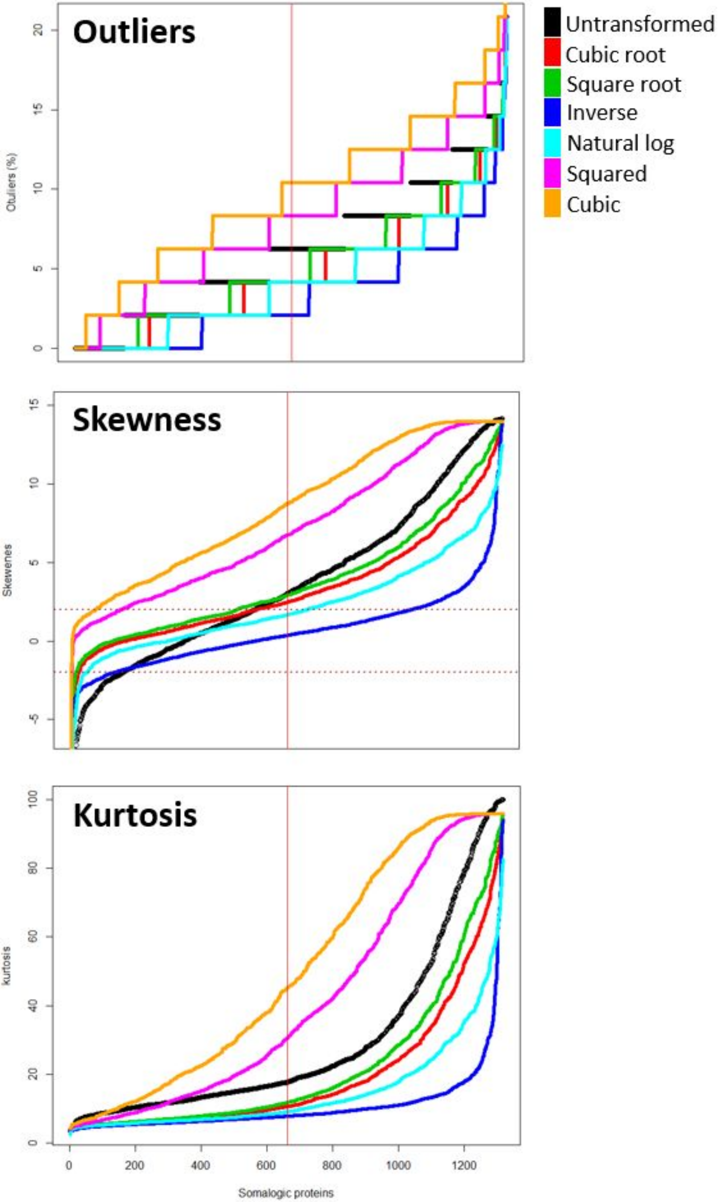

Outliers
Untransformed
Cubic root
Square root
Inverse
Natural log
Squared
Cubic
Otuliers (%)
Skewness
Skewenes
Kurtosis
kurtosis
Somalogic proteins
